# Mitocondrial COI and 16S rDNA sequences support morphological identification and biogeography of deep-sea red crabs of the genus *Chaceon* (Crustacea, Decapoda, Geryonidae) in the Eastern Central and South Atlantic Ocean

**DOI:** 10.1371/journal.pone.0211717

**Published:** 2019-02-11

**Authors:** Mariano Hernández, M. Virginia Martín, Pedro M. Herrador-Gómez, Sebastián Jiménez, Carlos Hernández-González, Santiago Barreiro, Roberto Sarralde, Barend Johannes van Zyl, Johny Charles Gamatham, Teresa Almeida, Luis J. López-Abellán

**Affiliations:** 1 Departamento de Bioquímica, Microbiología, Biología Celular y Genética, Instituto Universitario de Enfermedades Tropicales y Salud Pública de Canarias, Universidad de La Laguna, La Laguna, Santa Cruz de Tenerife, Spain; 2 Centro Oceanográfico de Canarias, Instituto Español de Oceanografía, Santa Cruz de Tenerife, Spain; 3 South East Atlantic Fisheries Organisation (SEAFO), Swakopmund, Namibia; 4 Ministry of Fisheries and Marine Resources, Swakopmund, Namibia; Hokkaido University, JAPAN

## Abstract

The geographical spreading of new fishing activities and the increasingly deeper locations of these activities have shown the worldwide distribution of gerionid crabs and new descriptions of *Chaceon* taxa. However, incomplete penetrance, variable expressivity, and phenotypic overlap make the morphometric identification of these species difficult. In this study, partial sequences of the cytochrome c oxidase subunit 1 (*COI*) and 16S mitochondrial ribosomal RNA (16S rRNA) genes have been analyzed in *Chaceon* species from the Eastern Central and South Atlantic and compared with sequences of species from Western Atlantic. Our results corroborate the proposed morphological species and highlight the significant separation of the Eastern Atlantic species and those from Atlantic coasts of South America for both markers (97% Bayesian posterior probability, BPP / 83% Bootstrap replicates, BT). Interestingly, *Chaceon sanctaehelenae* shows a closer relationship with the species of the American coast than with those from the Eastern Atlantic. On the other hand, while *COI* marker clearly separates *Chaceon atopus* and *Chaceon erytheiae* species (99 BPP / 91% BT), these species share haplotypes for the 16S rRNA marker, pointing to a recent speciation process. Moreover, a close relationship was observed between *Chaceon maritae* and *Chaceon affinis* (94% BPP / 77% BT). The topologies of the trees obtained indicate that the ancestor of this genus was closer related to those species from South America than to those from the Eastern Atlantic.

## Introduction

Morphological identification of species belonging to the Geryonidae family has been a difficult task in the past for people studying deep-sea resources and marine biodiversity. Interspecific similarities and intraspecific morphological variability have contributed to former and potential current misclassification [1]. Thus, other considerations such as the geographical site of collections have also been an element used in the species identification, regarding previous species citations in the same geographic zone [1, 2, 3].

Until the early eighties most species along the East Atlantic Ocean and also off the African coast of the Indian Ocean were misidentified as *Geryon quinquedens*, now a species restricted to the Northwest Atlantic. In 1981, Manning and Holthuis [2] described *Geryon maritae* as a new species in the Atlantic coast of Africa. These authors reported that most records of *Geryon affinis* or *Geryon quinquedens* from West Africa pertained to *G*. *maritae*.

Later, the geographical spreading of new exploratory fishing activities and associated studies, as well as the increasingly deeper locations of these fishing activities, provided new biological material that showed the worldwide distribution of gerionids and new species descriptions. Through this process, Manning and Holthuis [3] recorded three species of *Geryon* from South West Africa-Namibia and South Africa: *Geryon chuni*, *Geryon macphersoni* and *G*. *maritae*. Afterward, they faced the restructuration of the Geryonidae family moving most of the species in the genus *Geryon* into a new genus, *Chaceon* and describing nine new species [1].

Thus, since the eighties the number of new descriptions of *Chaceon* genus has considerably increased reaching the amount of 34 species [4]. However, genera of the Geryonidae family and some species assignment to these genus have been in controversy up to now. Thus, Ng et al. [5] listed 31 species of *Chaceon* where *C*. *sanctaehelenae* is dropped and *Chaceon chuni* and *Chaceon inghami* are considering as *Geryon chuni* and *Geryon inghami*.

In some cases, the multigenic inheritance, the reduced penetrance and variable expressivity of morphological traits, as well as the observer subjectivity could make the morphometric identification difficult. In this context, where the presence of cryptic species has high possibility to occur, molecular analysis could shed light on morphological identification and biogeography of the genus *Chaceon*.

In this sense, the use of 16S mitochondrial ribosomal RNA (16S rRNA) and cytochrome c oxidase subunit 1 (*COI*) allowed reconstructing the initial phylogenies in crustacean [6, 7]. Specific studies on *Chaceon* species have been conducted by Weinberg and col. [8] in the North Atlantic, and Mantelatto and col. [9] in the South West Atlantic. However, there are no studies providing a broader vision of phylogenetic relationships among species of *Chaceon* genus from Eastern Central and South Atlantic.

Therefore, the objective of this study is to review the morphology-based taxonomic classificaton of deep-sea red crabs of the genus *Chaceon* using mitocondrial COI and 16S rRNA sequences.

## Material and methods

**Fig 1:**
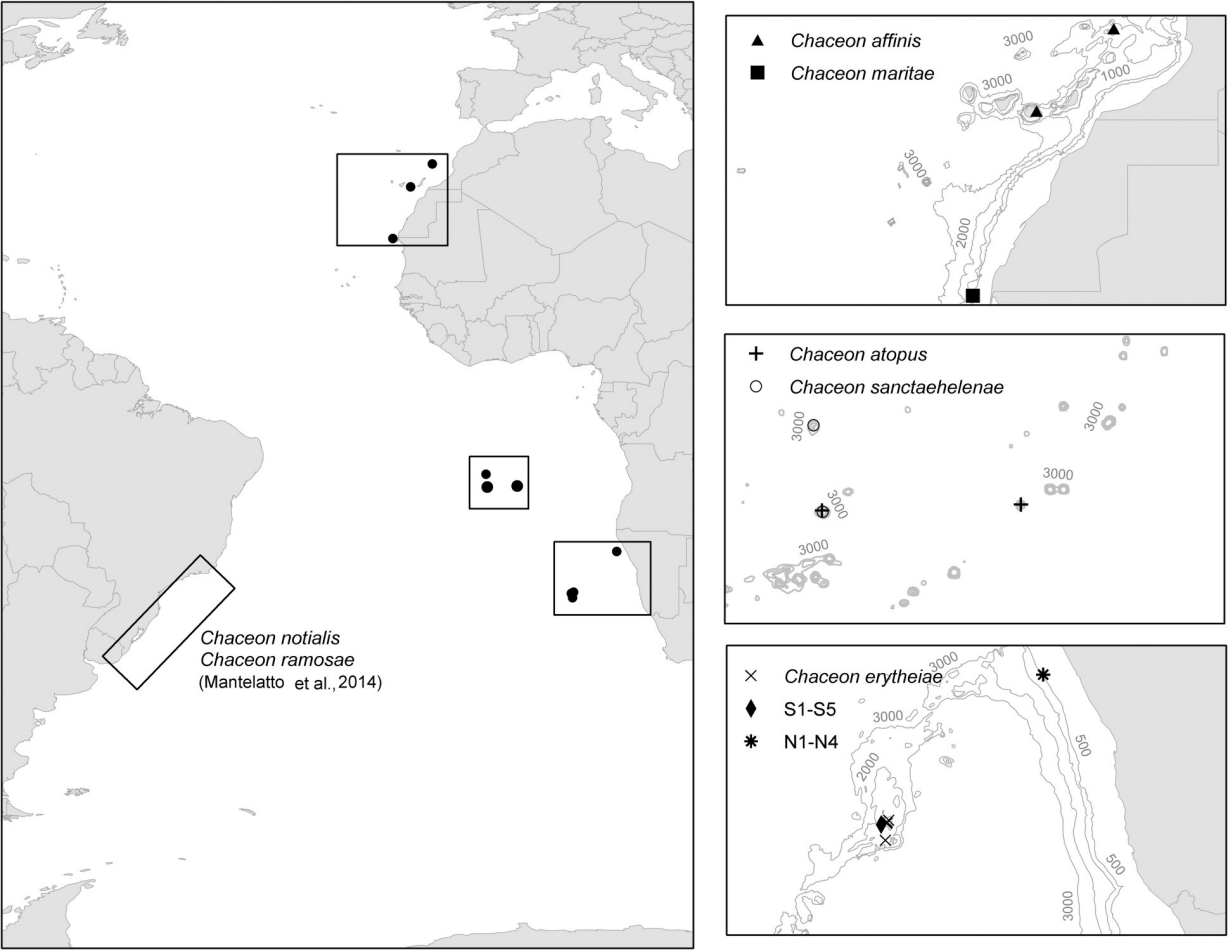
Geographical distribution of samples of *Chaceon* species collected at different locations of the Eastern Central and South Atlantic and approximate distribution area of South American species [9] used to contrast our results (maps enlarged to the right).

**Fig 2:**
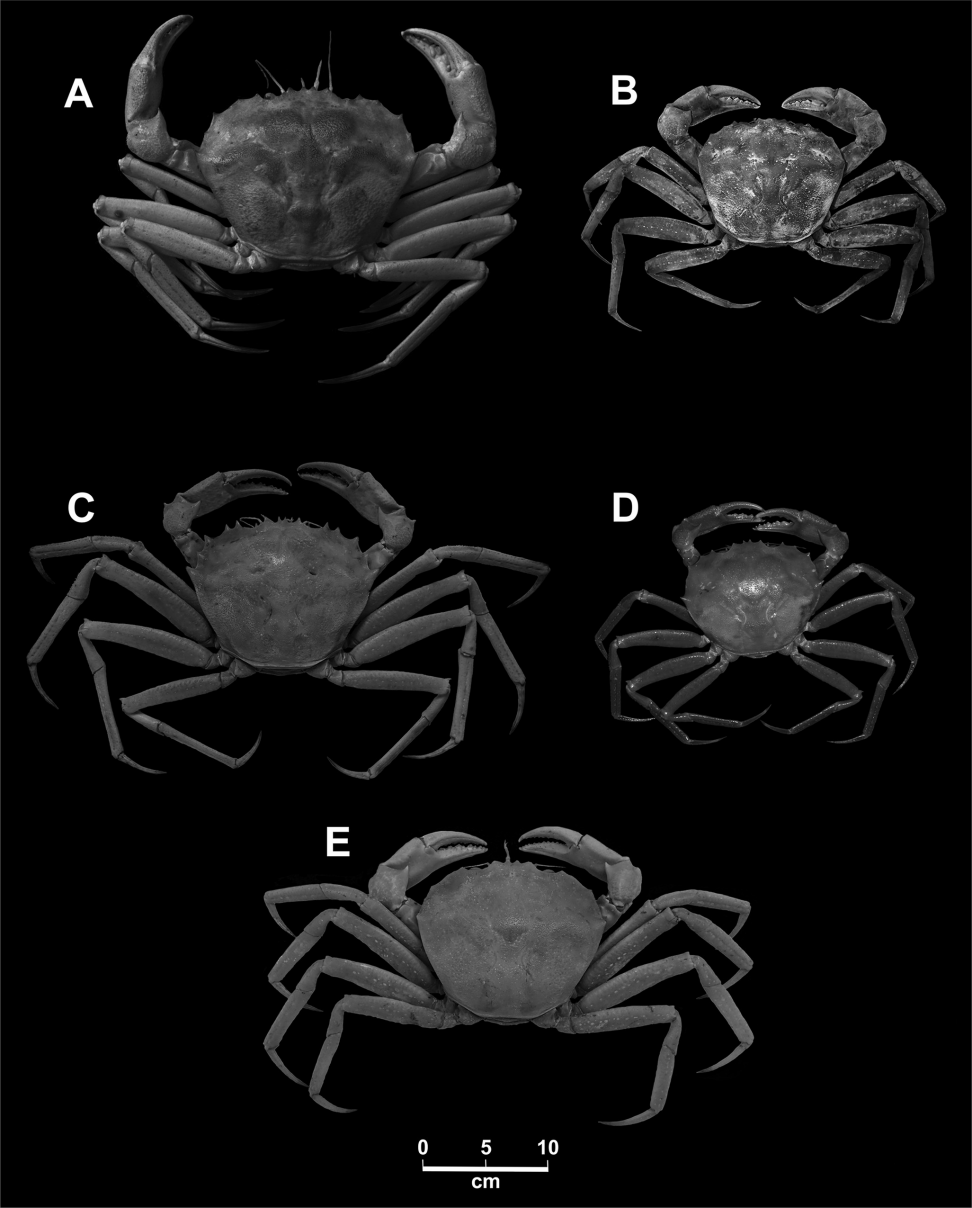
Specimens of five species of red crabs of the genus *Chaceon* (A) *C*. *affinis* [11], male carapace width 177 mm; (B) *C*. *maritae* [11] male carapace width 117 mm: (C) *C*. *atopus* [1], male carapace width 153 mm; (D) *C*. *erytheiae* [10], male carapace width 112 mm; and (E) *C*. *sanctaehelenae* [1], male carapace width 150 mm.

Forty-three samples of muscle tissue from individuals of deep-sea red crabs (*Chaceon* spp.) were collected from different places in the Eastern Central and South Atlantic (Fig 1). Species names assigned on morphological basis [1, 3, 10] and sampling areas are reported in Table 1. The presence and potential geographic distribution of species of *Chaceon* genus in the Central East and South Atlantic is shown in Fig 1. Five species of *Chaceon* were morphologically identified. *C*. *erytheiae* from Valdivia Bank was identified following Macpherson [10]. *C*. *atopus* [1] and *C*. *sanctaehelenae* [1] specimens collected in the Vavilov Ridge (Gulf of Guinea) were identified using Manning and Holthuis [1] description. Finally *C*. *affinis* [11] from the Canary Islands and Fantasma Bank (north Canary Islands) and *C*. *maritae* [2] from Western Sahara waters were identified through Manning and Holthuis [2]. Also, nine samples of unidentified specimens from Namibian Exclusive Economic Zone (EEZ) and the Walvis Ridge (Valdivia Bank) were incorporated to the analysis (Table 1; Figs 1 and 2). Table 1 also includes the serial code; the ID Code used on labelling the samples; name of research cruises or fishing trips in which samples were obtained; collectors; places of harvesting and depths; and measurements of individuals and sex. All collected specimens belonged to commercial exploited species so there is no prohibition for their fishing. The fishing vessels and fishing research vessels where they were captured have the legal permits to carry out their fishing and research activities. This study did not involve endangered or protected species.

**Table 1:** List of samples used in the analysis including data related to collection process, locations and biological information.

Serial Code	ID Code	Species	Cruise / Fishing Trip^3^	Collector	Geographical Area	Latitude	Longitude	Depth (m)	Carapace width (mm)	Carapace length (mm)	Weight (g)	Sex
1	CRAB01	*Chaceon erytheiae*	NAMIBIA 0902	COC-IEO^1^	Valdivia Bank	-26.1847	6.008	1038	85.8	70.5	151	F
2	CRAB02	*Chaceon erytheiae*	NAMIBIA 0902	COC-IEO	Valdivia Bank	-25.5627	6.0505	888	102.6	88.1	336	M
3	CRAB03	*Chaceon atopus*	CRUPER 2004	COC-IEO	Vavilov Ridge	-11.458	-1.298	705	136	105	-	F
4	CRAB04	*Chaceon atopus*	CRUPER 2004	COC-IEO	Vavilov Ridge	-11.458	-1.298	705	158	126	-	M
5	CRAB05	*Chaceon atopus*	CRUPER 2004	COC-IEO	Vavilov Ridge	-11.458	-1.298	705	153	122	-	M
6	CRAB06	*Chaceon atopus*	CRUPER 2004	COC-IEO	Vavilov Ridge	-11.579	-5.247	496	156	118	-	M
7	CRAB07	*Chaceon sanctaehelenae*	CRUPER 2004	COC-IEO	Vavilov Ridge	-11.601	-5.233	427	117	96	-	F
8	CRAB08	*Chaceon sanctaehelenae*	CRUPER 2004	COC-IEO	Vavilov Ridge	-11.601	-5.233	427	123	103	-	M
9	CRAB09	*Chaceon sanctaehelenae*	CRUPER 2004	COC-IEO	Vavilov Ridge	-11.601	-5.233	427	150	130	-	M
10	CRAB10	*Chaceon sanctaehelenae*	CRUPER 2004	COC-IEO	Vavilov Ridge	-9.886	-5.412	338	107	84	-	F
11	CRAB11	*Chaceon sanctaehelenae*	CRUPER 2004	COC-IEO	Vavilov Ridge	-9.886	-5.412	338	117	93	-	F
12	CRAB12	*Chaceon sanctaehelenae*	CRUPER 2004	COC-IEO	Vavilov Ridge	-9.886	-5.412	338	130	109	-	F
13	CRAB13	*Chaceon maritae*	MAROC 0611	COC-IEO	Western Sahara waters	21.179	-17.703	560	117	104	-	M
14	CRAB14	*Chaceon affinis*	GRAN CANARIA 1403	COC-IEO	Canary Is. (Taliarte)	27.99	-15.3556	750	123	103	463	M
15	CRAB15	*Chaceon affinis*	GRAN CANARIA 1403	COC-IEO	Canary Is. (Taliarte)	27.99	-15.3556	750	135	112	621	M
16	CRAB16	*Chaceon affinis*	GRAN CANARIA 1403	COC-IEO	Canary Is. (Taliarte)	27.99	-15.3556	750	123	101	493	M
17	CRAB17	*Chaceon affinis*	GRAN CANARIA 1403	COC-IEO	Canary Is. (Taliarte)	27.99	-15.3556	750	140	121	684	M
18	CRAB18	*Chaceon erytheiae*	NAMIBIA 0902	COC-IEO	Valdivia Bank	-26.1847	6.008	1038	77.3	66.1	126	F
19	CRAB19	*Chaceon erytheiae*	NAMIBIA 0902	COC-IEO	Valdivia Bank	-26.1847	6.008	1038	121.9	99.7	480	M
20	CRAB20	*Chaceon erytheiae*	NAMIBIA 0902	COC-IEO	Valdivia Bank	-26.1847	6.008	1038	116.8	98.9	466	M
21	CRAB21	*Chaceon erytheiae*	NAMIBIA 0902	COC-IEO	Valdivia Bank	-25.4466	6.1256	882	76.8	63.9	130	M
22	CRAB22	*Chaceon erytheiae*	NAMIBIA 0902	COC-IEO	Valdivia Bank	-25.5627	6.0505	888	90.3	77.5	194	F
23	CRAB23	*Chaceon erytheiae*	NAMIBIA 0902	COC-IEO	Valdivia Bank	-26.1847	6.008	1038	111.6	92	392	M
24	CRAB24	*Chaceon erytheiae*	NAMIBIA 0902	COC-IEO	Valdivia Bank	-25.5627	6.0505	888	78.4	64.5	131	F
25	CRAB25	*Chaceon erytheiae*	NAMIBIA 0902	COC-IEO	Valdivia Bank	-25.5128	6.0629	874	117.52	96.71	447	M
26	CRAB26	*Chaceon affinis*	MARANSA 012M8	COC-IEO	Fantasma Bank	31.0249	-12.511	399	123	99	-	M
27	CRAB27	*Chaceon erytheiae*	NAMIBIA 0902	COC-IEO	Valdivia Bank	-26.1847	6.008	1038	113	94.4	413	M
28	CRAB28	*Chaceon erytheiae*	NAMIBIA 0902	COC-IEO	Valdivia Bank	-25.5627	6.0505	888	102.3	86.8	300	M
29	CRAB29	*Chaceon erytheiae*	NAMIBIA 0902	COC-IEO	Valdivia Bank	-25.5627	6.0505	888	106.7	88.9	376	M
30	CRAB30	*Chaceon erytheiae*	NAMIBIA 0902	COC-IEO	Valdivia Bank	-26.1847	6.008	1038	108	92.2	372	M
31	CRAB31	*Chaceon erytheiae*	NAMIBIA 0902	COC-IEO	Valdivia Bank	-26.1847	6.008	1038	108.1	92	371	M
32	CRAB32	*Chaceon erytheiae*	NAMIBIA 0902	COC-IEO	Valdivia Bank	-26.1847	6.008	1038	81.5	66.1	121	F
33	CRAB33	*Chaceon erytheiae*	NAMIBIA 0902	COC-IEO	Valdivia Bank	-26.1847	6.008	1038	109.6	92.8	378	M
34	CRAB34	*Chaceon erytheiae*	NAMIBIA 0902	COC-IEO	Valdivia Bank	-25.5627	6.0505	888	93.7	79.4	214	F
35	CRAB35	*S1*	SEAFO Comm. Trip	JG-BVZ^2^	SEAFO (B1)	-25.6035	5.8437	1087	102	84	232	M
36	CRAB36	*S2*	SEAFO Comm. Trip	JG-BVZ	SEAFO (B1)	-25.6035	5.8437	1087	102	80	250	F
37	CRAB37	*S3*	SEAFO Comm. Trip	JG-BVZ	SEAFO (B1)	-25.6035	5.8437	1087	116	92	355	F
38	CRAB38	*S4*	SEAFO Comm. Trip	JG-BVZ	SEAFO (B1)	-25.6035	5.8437	1087	112	89	272	F
39	CRAB39	*S5*	SEAFO Comm. Trip	JG-BVZ	SEAFO (B1)	-25.6035	5.8437	1087	111	91	335	M
40	CRAB40	*N1*	NAMIBIA Comm. Trip	JG-BVZ	Namibia EEZ	-20.074	11.815	458	119	105	675	M
41	CRAB41	*N2*	NAMIBIA Comm. Trip	JG-BVZ	Namibia EEZ	-20.074	11.815	458	128	113	828	M
42	CRAB42	*N3*	NAMIBIA Comm. Trip	JG-BVZ	Namibia EEZ	-20.074	11.815	458	140	121	906	M
43	CRAB43	*N4*	NAMIBIA Comm. Trip	JG-BVZ	Namibia EEZ	-20.074	11.815	458	122	103	504	M

1) Centro Oceanográfico de Canarias—Instituto Español de Oceanografía

2) Namibian collectors

3) Name of research cruises or fishing trips

The forty-three samples of muscle tissue collected were preserved in absolute ethanol at 4°C until their analysis. Total genomic DNA was extracted, using a standard phenol/chloroform extraction procedure [12] after an overnight proteinase K digestion (20 mg/ml) at 56°C. Two fragments of mitochondrial genes, 16S ribosomal DNA (16S rDNA) and cytochrome oxidase (COI), were amplified using primer pairs 16Sar [13]; 16Sbr.crab [8]; CoL6b [14]; and COIF.crab (5’CgTgCTgAACTTggTCAAC-3’) (this study), respectively.

Amplifications of PCR were performed in a Bio-Rad S1000 Thermal Cycler with dual bloc in a total volume of 25 μl including 1x buffer (GeneAll Biotechnology, South Korea), 150 μM of each dNTP, 0.4 μM of each primer, 1 U of AmpOne Taq DNA polymerase (GeneAll Biotechnology, South Korea) and 20 ng of total genomic DNA. PCR conditions were as follows: 2 min at 94°C followed by 35 cycles of denaturation at 94°C for 10 s, annealing at 54°C for 20 s, and extension at 72°C for 30 s, with a final extra extension step at 72°C for 10 min.

Reaction efficiencies were estimated by electrophoresis in agarose real-safe stained gels (Real Biotech Corporation, Taiwan). Subsequently, PCR products were enzymatically purified with Illustra ExoProStar 1-Step (GE Healthcare, UK) according to the manufacturer’s instructions. Sequencing reactions were performed for both strands with the same primers used in the amplification at the Genomic Service of the University of La Laguna (SEGAI). Sequences of DNA were edited and assembled using MEGA v. 6 [15]. Sequence alignment was performed using CLUSTAL W [16] as implemented in MEGA. The absence of stop codons in the COI alignment was confirmed using the software MEGA 6 [15]. In order to establish the phylogenetic relationships within the *Chaceon* genus, we used published sequences from other two *Chaceon* species, *C*. *notialis* and *C*. *ramosae* [9] from the Western Central Atlantic, as well as those from *Liocarcinus depurator* and *Carcinus aestuarii* used as outgroups in the analysis. Accession numbers to GenBank data base of all sequences used are reported in Table 2.

**Table 2:** GenBank accession numbers of mtDNA COI and 16S rRNA gene sequences.

COI					16S rRNA			
Species	Haplotypes	GenBank accession numbers		Number of individuals	Species	Haplotypes	GenBank accession numbers	Number of individuals
*C*. *erytheiae*	1	LN809890		1	*C*. *erytheiae*	1	LN809917	22
*C*. *erytheiae*	2	LN809891		1				
*C*. *atopus*	1	LN809892		2				
*C*. *atopus*	2	LN809893		1				
*C*. *atopus*	3	LN809894		1				
*C*. *sanctaehelenae*	1	LN809895		1	*C*. *sanctaehelenae*	1	LN809918	5
*C*. *sanctaehelenae*	2	LN809896		1	*C*. *sanctaehelenae*	2	LN809919	1
*C*. *sanctaehelenae*	3	LN809897		1				
*C*. *maritae*	1	LN809898		2	*C*. *maritae*	1	LN809920	4
*C*. *affinis*	1	LN809899		1	*C*. *affinis*	1	LN809921	2
*C*. *affinis*	2	LN809900		1	*C*. *affinis*	2	LN809922	1
*C*. *affinis*	3	LN809901		1	*C*. *affinis*	3	LN809923	1
*C*. *affinis*	4	LN809902		1				
*C*. *erytheiae*	3	LN809903		1	*C*. *erytheiae*	2	LN809924	1
*C*. *erytheiae*	4	LN809904		1	*C*. *erytheiae*	3	LN809925	1
*C*. *erytheiae*	5	LN809905		2	*C*. *erytheiae*	4	LN809926	1
*C*. *erytheiae*	6	LN809906		8	*C*. *erytheiae*	5	LN809927	1
*C*. *erytheiae*	7	LN809907		1	*C*. *erytheiae*	6	LN809928	1
*C*. *affinis*	5	LN809908		1				
*C*. *erytheiae*	8	LN809909		1				
*C*. *erytheiae*	9	LN809910		1				
*C*. *erytheiae*	10	LN809911		1				
*C*. *erytheiae*	11	LN809912		1				
*C*. *erytheiae*	12	LN809913		1				
*C*. *erytheiae*	13	LN809914		1				
*C*. *maritae*	2	LN809915		1	*C*. *maritae*	2	LN809929	1
*C*. *maritae*	3	LN809916		2				
*C*. *ramosae* 1	-		KC676772	-				*-*
*C*. *ramosae* 2	-		KC676777	-	*C*. *ramosae* 2	*-*	KC676755	
*C*. *notialis* 1	-		KC676760	-	*C*. *notialis* 2	*-*	KC676731	*-*
*C*. *notialis* 2	-		KC676762	-	*C*. *notialis* 3	*-*	KC676732	*-*
*C*. *notialis* 3	-		KC676763	-				
**Outgroups**								
*Liocarcinus depurator*	-		JQ305906	-	*Liocarcinus depurator*	*-*	FM208767	*-*
*Carcinus aestuarii*			KC311390		*Carcinus aestuarii*		U74327	

Site saturation was evaluated in DAMBE v.5.5.18 software [17] by plotting the numbers of observed transitions and transversions against pairwise genetic distance.

Phylogenetic relationships were constructed from both individual mitochondrial COI and 16S rDNA, as well as concatenated sequences. The best-fit nucleotide substitution model was determined using jModelTest 2 [18] according to the Bayesian Information Criterion. HKY + G and HKY + I, were the chosen models for COI and 16S rDNA, respectively and so used in the Maximum Likelihood (ML) and Bayesian Inference (BI) analyses. In the combined data set, substitution models, parameters of nucleotide frequencies, substitution rates, gamma shape, and invariant sites proportion were unlinked across partitions.

ML was performed with Treefinder version March 2011 [19] always with 1,000 Bootstrap replicates (BT). BI analysis was performed using MrBayes v.3.2.1 [20] on the Mobyle SNAP Workbench [21]. Two parallel runs were performed in MrBayes using four Markov chain Monte Carlo (MCMC) chains. Ten million MCMC generations were run, with a sampling frequency every 10^2^ generations. Of the resulting trees, the first 10,001 trees were discarded as burn in after checking for stationarity with TRACER v1.3 [22], and the following 90,000 trees were used to estimate the topology and tree parameters. Percentage of times a node occurred within those 90,000 trees was interpreted as the Bayesian posterior probability (BPP) of the node. Trees generated from both analyses were edited by Treegraph 2 [23].

## Results

From all 43 samples of deep-sea red crabs (*Chaceon* spp.), a fragment of 468 base pairs (bp) of mtDNA COI gene was amplified and sequenced from 38 samples. The absence of stop codons seems to discard the presence of nuclear mitochondrial insertions (Numts) in the sequences. Among the 468 nucleotide sites, 142 sites were variable and 87 were phylogenetically informative. From 42 samples, the amplification of the corresponding fragment of 16S rRNA resulted in a single band of 429–430 bp. The alignment of the sequences generated a 432 bp matrix because of the need to include "gaps" (variants insertion / deletion, "indels") for optimal alignment. Of the 432 sites, 104 showed variation and of these, 47 were informative. Indels were discarded in subsequent analyses. The absence of double bands in PCR and double peaks in the electropherogram of the sequences as well as the similarity among them and with those originals deposited in Genbank seems to discard the presence of nuclear mitochondrial DNA sequences (Numts) in these sequences too.

**Fig 3:**
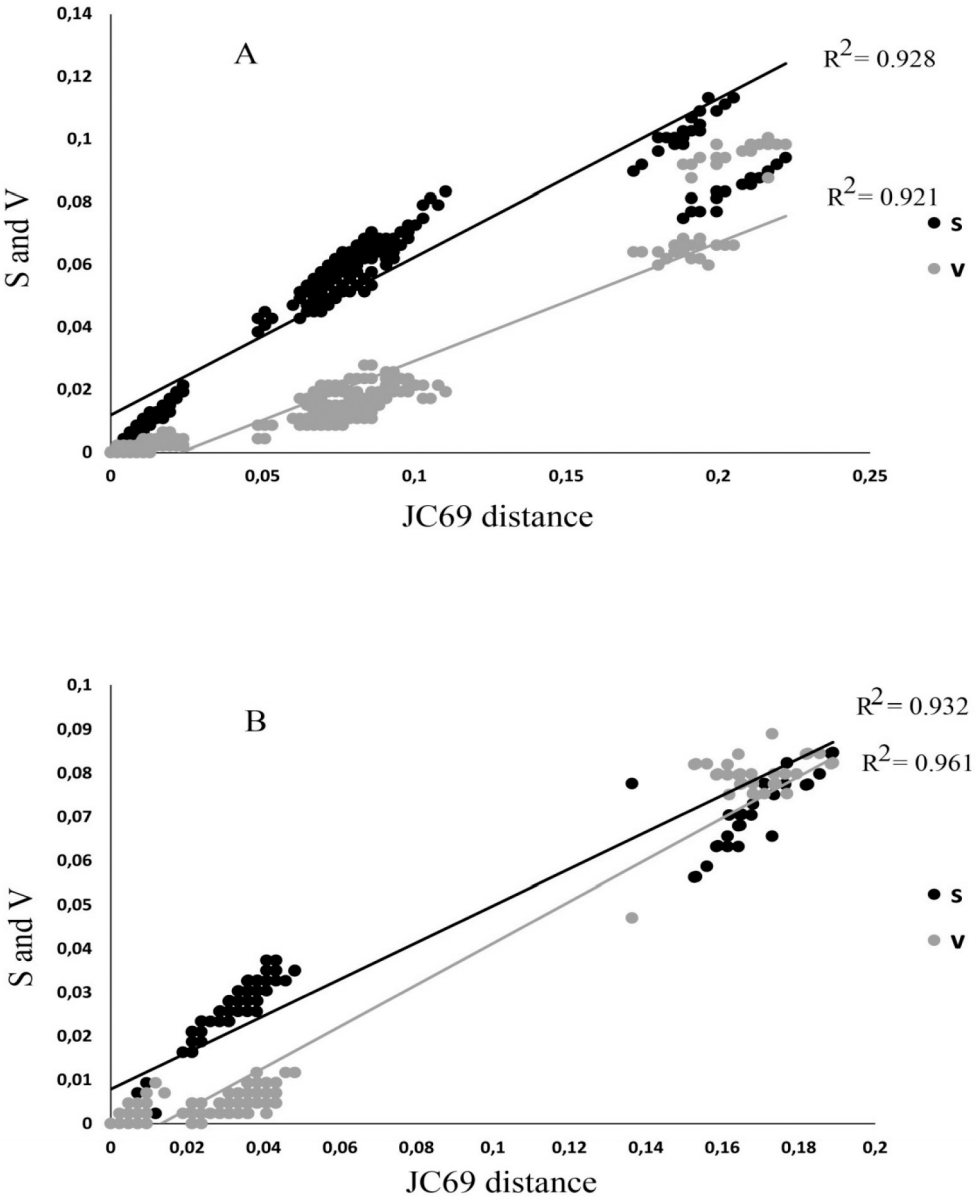
Analysis of substitutions (transitions and transversions) for the COI (a) and 16S rRNA (b) data set from species of Chaceon using JC69 distances (%).

**Fig 4:**
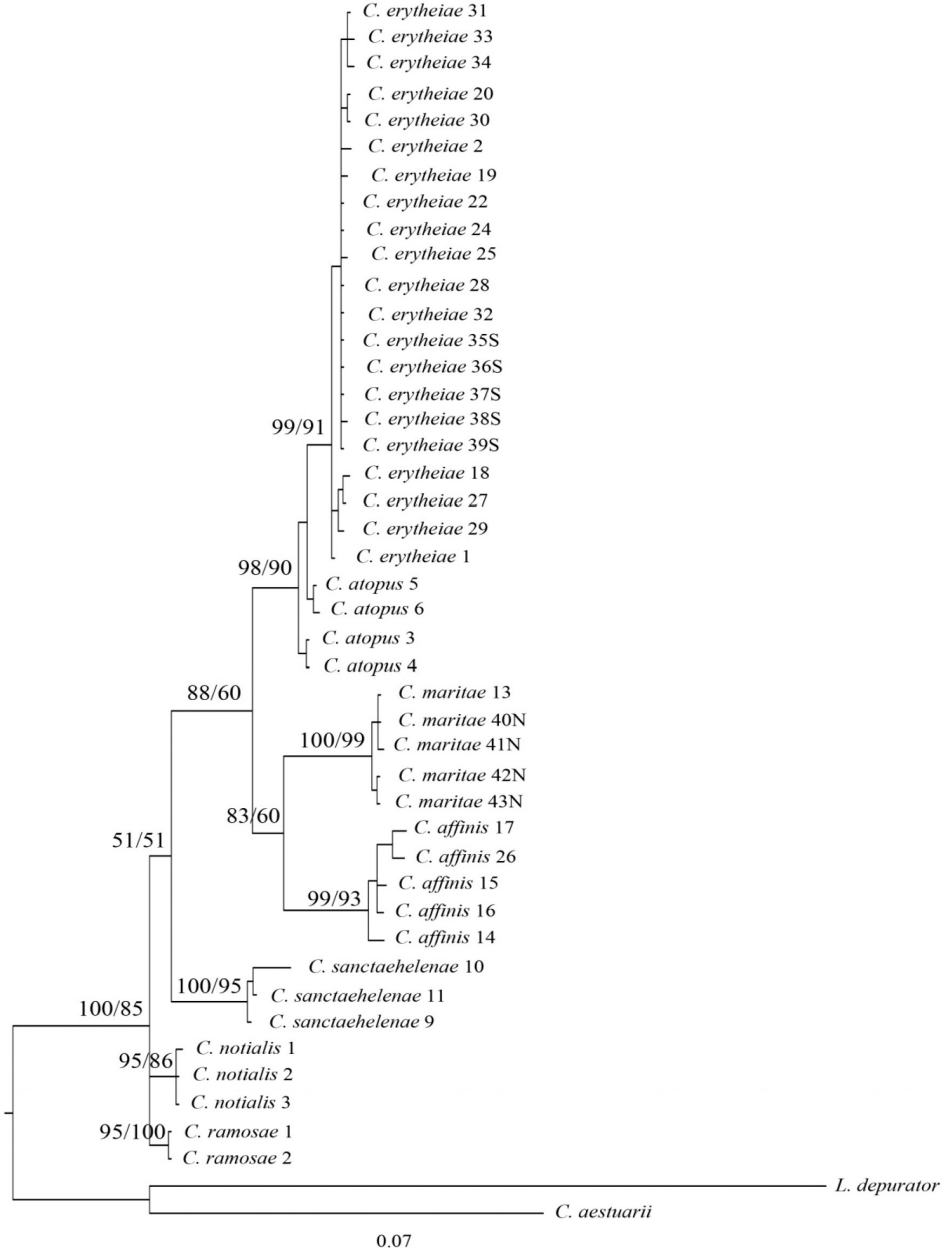
Bayesian phylogenetic tree showing the relationships among species of Chaceon based on a 468 bp COI fragment. Numbers after species name correspond with the serial code in Table 1 except for *C*. *notialis* and *C*. *ramosae*. Numbers at nodes represent Bayesian posterior probability and Bootstrap values of maximum likelihood analysis, respectively.

**Fig 5:**
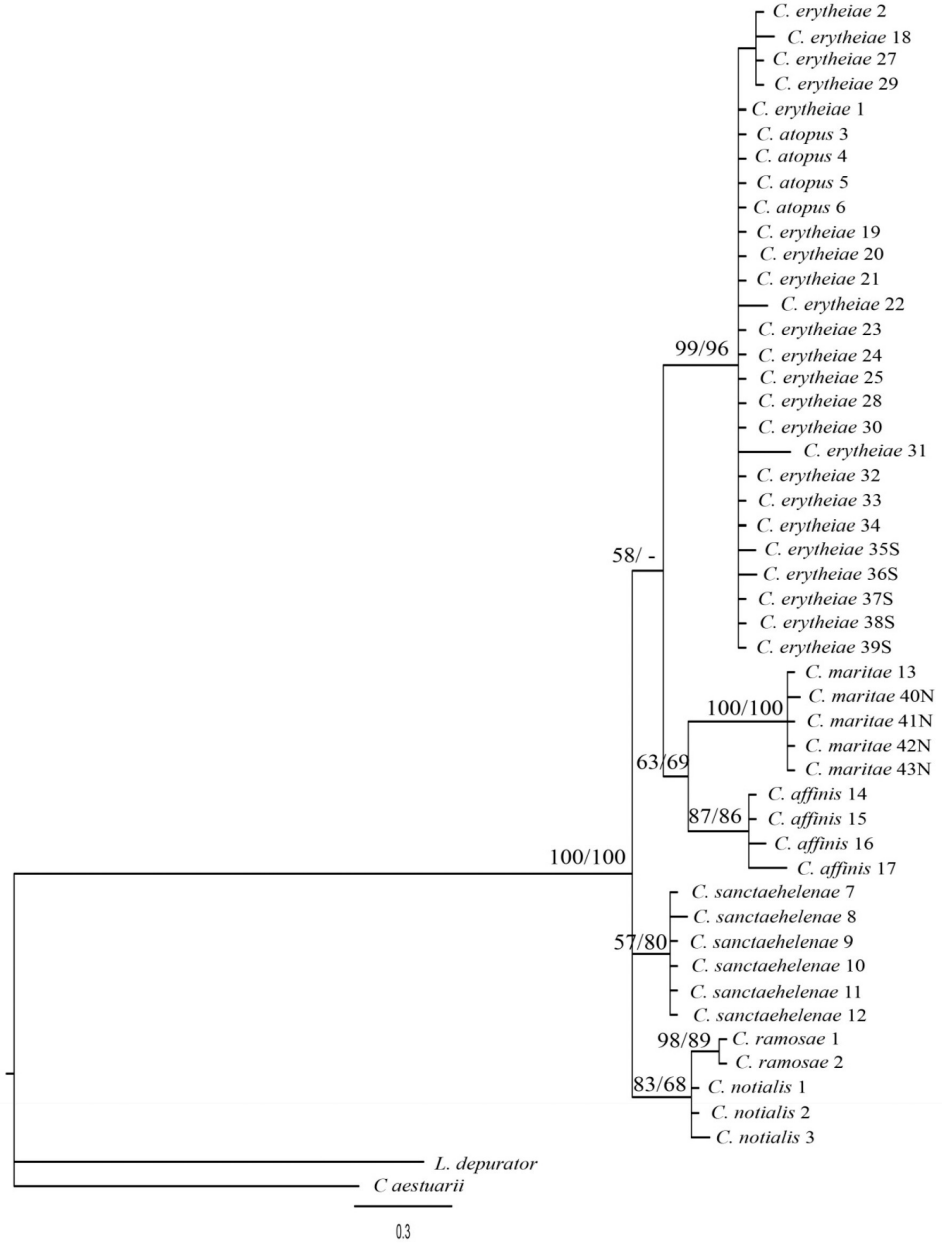
Bayesian phylogenetic tree showing the relationships among species of Chaceon based on a 432 bp 16S rRNA fragment. Numbers after species name correspond with the serial code in Table 1 except for *C*. *notialis* and *C*. *ramosae*. Numbers at nodes represent Bayesian posterior probability and Bootstrap values of maximum likelihood analysis, respectively.

**Fig 6:**
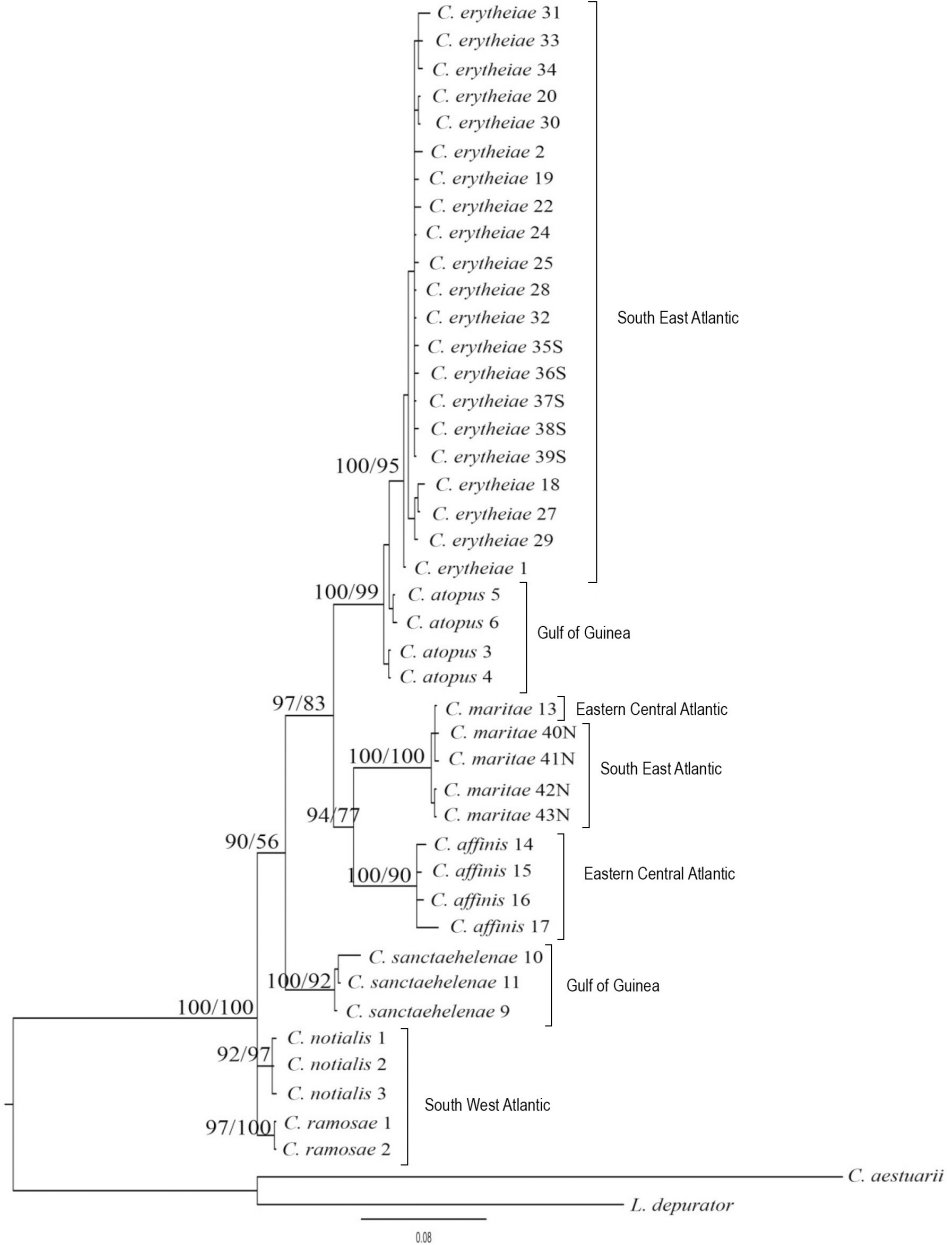
Bayesian phylogenetic tree showing the relationships among species of Chaceon based on the combined data set of COI and 16S rRNA genes. Numbers after species name correspond with the serial code in Table 1 except for *C*. *notialis* and *C*. *ramosae*. Numbers at nodes represent Bayesian posterior probability and Bootstrap values of maximum likelihood analysis, respectively.

The detected variation between sequences showed no deviation from neutrality in either case (D = -1.23 and D = -0.31 with P> 0.10 for 16S rRNA and COI, respectively). The saturation plots for COI and 16S rRNA (Fig 3) show that transitions and transversions are roughly linearly correlated, with the Jukes-Cantor genetic distance [24] with no obvious tendency to level off. In addition, Xia`s index for substitution saturation in *COI* produced values of 0.024 (first and second codon positions) and 0.104 (third codon position), and 0.067 in the case of the 16S rRNA, significantly lower (P < 0.001 in all cases) than the critical values for symmetric topology. Phylogenetic trees obtained by both methods showed the same topology and so, only Bayesian trees are shown in Figs 4, 5 and 6 for COI, 16S rRNA, and for the combination of both, respectively. The Bayesian posterior probability (BPP) and Bootstrap values (BT), supporting the clades are indicated on branches.

The first point that emerges from these relationships is the significant separation of Eastern Atlantic species, excluding *C*. *sanctaehelenae*, from those who inhabit the Atlantic coasts of the American continent when we take into account the phylogenetic tree for the concatenated sequences (97% BPP / 83% BT) (Fig 6). Curiously, *C*. *sanctaehelenae* species shows a closer relationship with the species of the American coast, *C*. *ramosae* and *C*. *notialis*, than with those from the Eastern Atlantic as *C*. *atopus*, although the distribution areas of both species overlap. On the other hand, all analyses strongly support the close relationship between *C*. *atopus* and *C*. *erytheiae* species (100% PPB / 99% BT) (Fig 6). However, while COI marker clearly separates both species (99% BPP / 91% BT) (Fig 4), individuals from *C*. *atopus* and *C*. *erytheiae* share haplotypes for the 16S rRNA marker (Fig 5).

Finally, it is worth noting that the sequences of 9 samples from Namibia which had not been morphologically identified turned out to belong to the species *C*. *erytheiae* (S1-S5) and *C*. *maritae* (N1-N4) (Table 1) as it was expected, given the geographical location of their capture (Fig 1). A close relationship was observed between *C*. *maritae* and *C*. *affinis* (94% BPP / 77% BT). Specimens of *C*. *maritae* from the Sahara Coast and Namibia waters did not show any differences between them and formed their own clade with 100% Bayesian posterior probability (Fig 6). On the other hand, *C*. *affinis* specimens were also grouped in a different clade with a relatively high branch support value (100% BPP / 90% BT) (Fig 6).

## Discussion

Mitochondrial DNA (mtDNA) is the marker commonly used for genetic reconstruction of the history of populations, population demography, biogeography and speciation, and recommended for taxonomic studies [25]. Despite the fact that mtDNA is not exempt from some disadvantages as the presence of Numts or pseudogenes [26], the presence of nuclear sequences is highly unlikely in our data.

Nowadays, only two phylogenetic studies have been found regarding *Chaceon* Atlantic species [8, 9] from the Northwest and Southwest Atlantic. Thus, the present work adds a new piece to the puzzle for the Southeast Atlantic.

Our results show that *Chaceon* species from the Central and South East Atlantic seem to have shared a common immediate ancestor with the Western Atlantic species.

Although it is difficult to contrast our results with those from other authors when different species are studied, some ideas can be extracted expanding the geographic scope. Weinberg et al. [8] analyzed samples from the Northwest Atlantic, *Chaceon quinquedens* (Southern New England and Gulf of Mexico) and *C*. *fenneri* (Eastern Florida). Three sequences of *C*. *affinis*, from the Eastern Atlantic (Madeira Islands and Canary Islands) were also included in the analysis. The authors detected genetic-based subdivision between *C*. *quinquedens* from the New England region of the Atlantic Ocean and *C*. *quinquedens* from the Gulf of Mexico by using sequence data from two genetic loci (16S and ITS). However, they found little or no genetic difference (16S data) between *C*. *quinquedens* from the Gulf of Mexico, *C*. *fenneri* from Eastern Florida, and *C*. *affinis* from the Eastern Atlantic, suggesting that these trans-Atlantic taxa share a more recent common history than the two populations of “*C*. *quinquedens*”.

Trying to compare our results with those of Weinberg et al. [8] we added all their sequences to our 16S rRNA data matrix. The output tree (S1 Fig) identified the samples of New England *C*. *quinquedens* as *C*. *erytheiae*, being their haplotypes identical. Also, the sequences of *C*. *quinquedens* from the Gulf of Mexico corresponded to sequences of *C*. *affinis* from the East Atlantic. In spite of the absence of COI sequences for those specimens, the coincidence of the phylogenetic trees for 16S RNAr and COI in our study would be in agreement with a misclassification.

In their article, Mantelatto et al. [9] showed that species like *C*. *ramosae* and *C*. *notialis* form a differentiated clade, aside from *C*. *affinis* and *C*. *maritae* clade and that the estimated genetic distance of COI gene among *Chaceon* specimens can be used to separate the species *C*. *notialis* and *C*. *ramosae*. Once this paper was published, we realized the possible incorrect geographical location of their *C*. *maritae* identification and we personally confirmed that these samples really belong to the Valdivia Bank. In this sense, given our research experience in this area we wonder whether the morphological identification of these specimens is correct. Although two species were initially identified for this region [27, 28], current reanalysis demonstrated the presence of one single species in all samples (*C*. *erytheiae*) belonging to the Valdivia Bank.

In this case, we also tried to understand similarities and differences between their analyses and our findings, adding their sequences to our data matrix and reanalyzing the outputs. The phylogenetic tree for COI (S2 Fig) showed that their *C*. *maritae* sequences have 100% similarity with our samples of *C*. *erytheiae*, reaffirming the possible morphological misidentification of this species by Mantelatto et al. [9]. It was not possible to carry out the same analysis with their sequences for 16S RNAr since these only overlap with our sequences 200 nts. Anyway, their sequences are identical to ours of *C*. *erytheiae* for the region shared. All the samples we analyzed from Valdivia Bank belong to *C*. *erytheiae*. However, we also have doubts about the COI sequence they obtained from GenBank for *C*. *affinis*, finally not published, and different to the ones that we obtained for this species (S2 Fig) which could explain the contradictory results they found between markers.

*C*. *maritae* is the main Geryonidae species in the West African coast, from the Western Sahara coast to the Namibian waters; it is located in the sandy-muddy continental slope at depths from 200 to 900 meters. This species is part of important fisheries (as by-catch in trawling deep-sea shrimp fisheries or target species in trap fisheries) along its distribution area from which Mauritania, Senegal, Angola or Namibia can be highlighted [29, 30, 31, 32, 33]. Results indicate that analyzed samples from Western Sahara coast and Namibian waters, both extremes of its broad geographic distribution, belong to the same species (BT and BPP = 100%), which constitutes a quite relevant fact taking into account the physical separation. However, this homogeneity could be explained by horizontal migratory processes of this species along the African coast. Records of mark and recapture individuals of this species reveal that mature females covered considerable distances of up to 380 km, but commonly between 100 and 180 km [28].

Despite being morphologically different, *C*. *maritae* forms a clade with *C*. *affinis*. *C*. *affinis* is a species that has colonized oceanic archipelagos from Cape Verde to Azores [34, 35, 36] and seamounts off the Central African coast and also the deepest continental slope (700–1900 m) off Morocco and Western Sahara (C. Hernández-González, IEO, pers. comm.), off the Atlantic coast of the Iberian Peninsula [37], Northeast Atlantic southern 64°N (Iceland) [38] and the Mid-Atlantic Ridge hydrothermal vents [39]. There are few records of the presence of these two species in the same archipelago or seamount, only in the Canary Islands and Cape Verde [40, 41, 42]. When more than one species settle in any geographic place, the depth range of distribution of each one tends to be different. This is the case of *C*. *affinis*, which distribution is deeper (500–1900 m) than *C*. *maritae* (100–950 m). Both species also show different patterns of size and sex population structure in relation to depth [35].

Similarly, *C*. *atopus* and *C*. *sanctaehelenae* seem to inhabit different depths in the same area of Vavilov Ridge, 500–800 m and 300–400 m, respectively. In relation to these two species it is interesting to remark that the ancestor of each one seems to have a different geographic origin, being that of *C*. *sanctaehelenae* in the Southwest Atlantic (*C*. *notialis* and *C*. *ramosae*); while the ancestor of *C*. *atopus* appears to have existed in the eastern Atlantic.

*C*. *atopus* and *C*. *erytheiae* share a preference for habitats deeper than 500 m, reaching depths near to 2000 m in the case of *C*. *erytheiae*. For these species, the molecular variation detected for COI, the marker with a higher substitution rate, allowed to distinguish haplotypes from both species, while for 16S rRNA marker individuals of both species shared haplotypes. This speaks in favour of a process of incipient speciation between these species.

Photographic records of *Chaceon* specimens caught along the Walvis Ridge, in the south-west Valdivia Bank, suggest the presence of *C*. *erytheiae* (size<140 mm of carapace width, CW) at those latitudes, until the Wust Seamount. Size records of individuals from this region and the southernmost Discovery Seamounts also suggest the presence of another bigger species that seems to be *Chaceon gordonae* (size<180 mm of CW), that is one of the cited species in the northern part of the region which we missed in our genetic analysis.

The basal position of species from the continental slope off Brazil and Uruguay seem to indicate a closer relationship with the ancestor of the genus. Consistent with our results, the presence of fossil *Chaceon* records (*C*. *peruvianus*) in South America [43] could represent the origin of current species in the Eastern Central and South Atlantic, in a parallel process of migration-dispersal and speciation northward and eastward. The most suitable path to cross the Atlantic would be the equatorial region, with a complex system of currents and countercurrents [44] at different depths that would help pelagic larvae transport [45] to new suitable habitats in the intermediate seamounts until reaching the west coast of Africa, spreading out northward and south by migrations and reaching new oceanic features by larvae transport. In this last mechanism, local oceanographic events as the Angola Gyre could have played an important role. Also features as the Mid-Atlantic Ridge, Walvis Ridge and Rio Grande Rise comprise potential paths that could explain the distribution diversity of *Chaceon* species in those regions.

## Supporting information

S1 Fig Bayesian phylogenetic tree showing the relationships among species of *Chaceon* based on a 432 bp 16S rRNA fragment, including the sequences obtained by Weinberg et al. (2003) for *C quinquedens* from Gulf of Mexico and New England. Numbers after species name correspond with the serial code in Table 1 except for *C*. *notialis* and *C*. *ramosae*. Numbers at nodes represent Bayesian posterior probability and Bootstrap values of maximum likelihood analysis, respectively.
(TIF)

S2 Fig Bayesian phylogenetic tree showing the relationships among species of *Chaceon* based on a 468 bp COI fragment, including the sequences obtained by Mantellato el al. (2014) for *C*. *maritae* and *C*. *affinis*. Numbers after species name correspond with the serial code in Table 1 except for *C*. *notialis* and *C*. *ramosae*. Numbers at nodes represent Bayesian posterior probability and Bootstrap values of maximum likelihood analysis, respectively.
(TIF)
