# Supplementary figures and images for: Mitocondrial COI and 16S rDNA sequences support morphological identification and biogeography of deep-sea red crabs of the genus Chaceon (Crustacea, Decapoda, Geryonidae) in the Eastern Central and South Atlantic Ocean
Source: PLoS One. 2019 Feb 11;14(2):e0211717. doi: 10.1371/journal.pone.0211717 (PMC6370203; doi:10.1371/journal.pone.0211717)

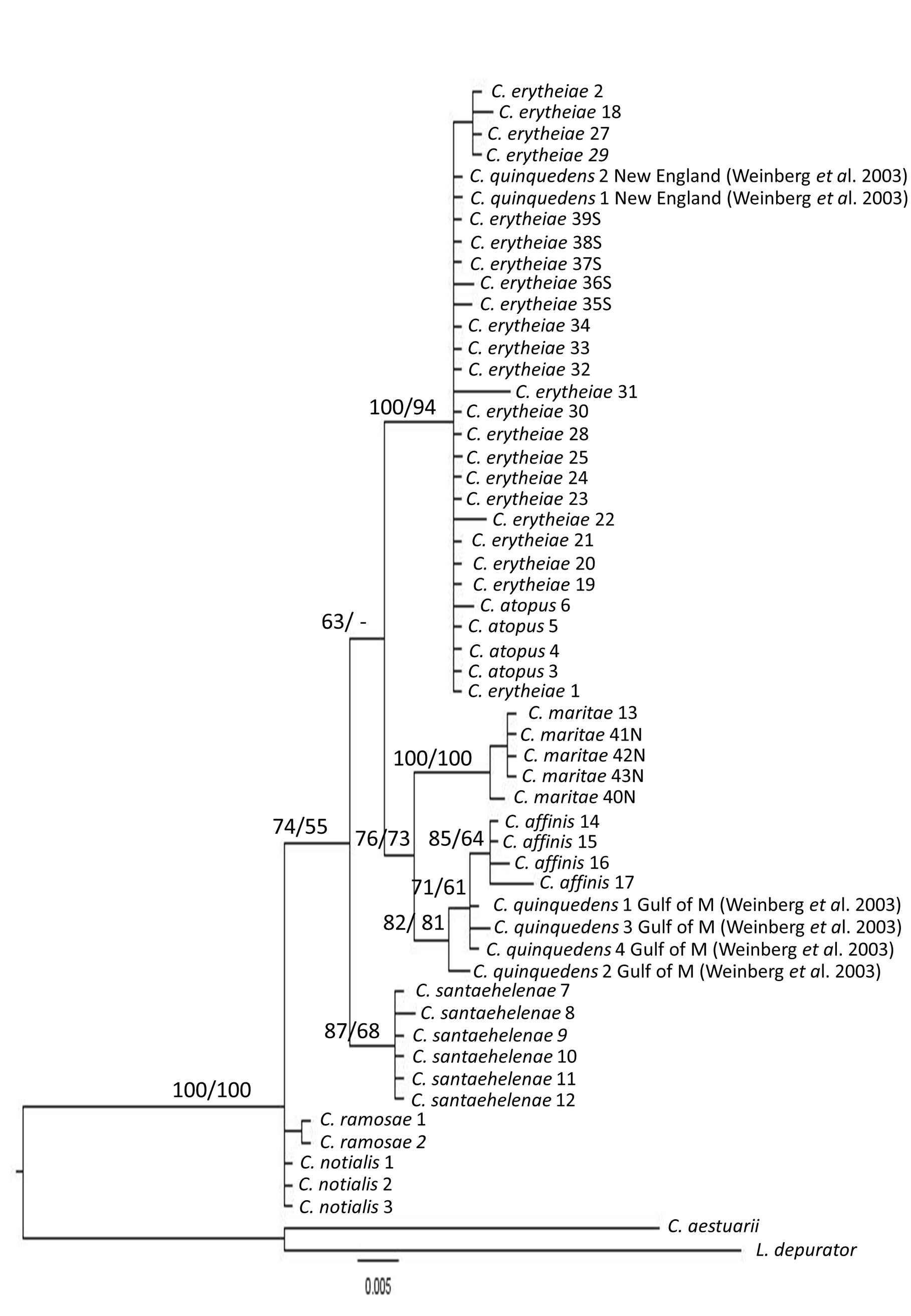

Supplement: S1 Fig — Numbers after species name correspond with the serial code in Table 1 except for C. notialis and C. ramosae. Numbers at nodes represent Bayesian posterior probability and Bootstrap values of maximum likelihood analysis, respectively. (TIF) [file pone.0211717.s001.tif]

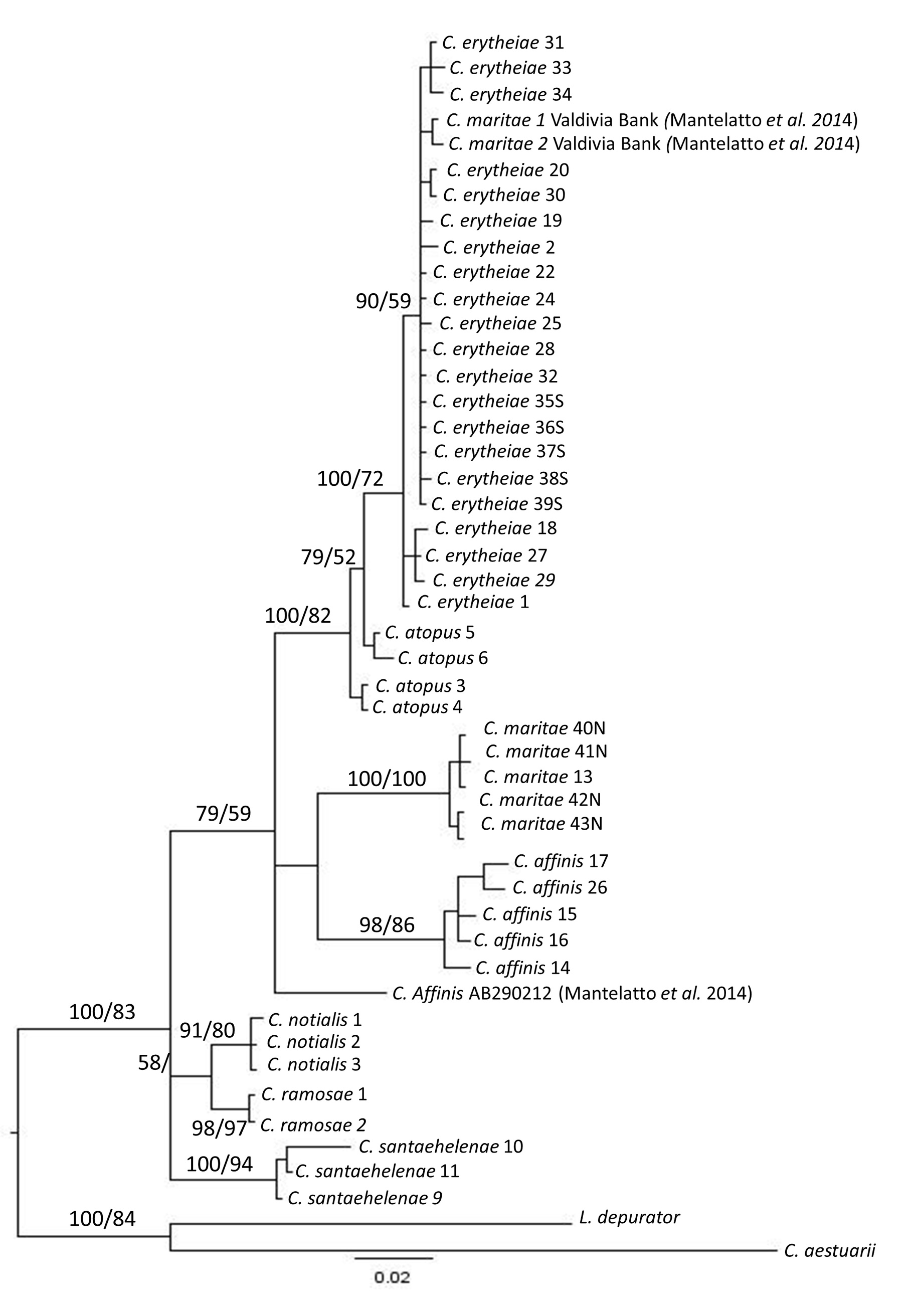

Supplement: S2 Fig — Numbers after species name correspond with the serial code in Table 1 except for C. notialis and C. ramosae. Numbers at nodes represent Bayesian posterior probability and Bootstrap values of maximum likelihood analysis, respectively. (TIF) [file pone.0211717.s002.tif]
